# Phospholipid imprinted polymers as selective endotoxin scavengers

**DOI:** 10.1038/srep44299

**Published:** 2017-03-17

**Authors:** Robert Sulc, Gyorgy Szekely, Sudhirkumar Shinde, Celina Wierzbicka, Filipe Vilela, David Bauer, Börje Sellergren

**Affiliations:** 1 Faculty of Chemistry, Technical University of Dortmund, Germany; 2 Hovione FarmaCiencia SA, R&D, Lisbon, Portugal; 3 Department of Biomedical Sciences, Faculty of Health and Society, Malmö University, Malmö, Sweden

## Abstract

Herein we explore phospholipid imprinting as a means to design receptors for complex glycolipids comprising the toxic lipopolysaccharide endotoxin. A series of polymerizable bis-imidazolium and urea hosts were evaluated as cationic and neutral hosts for phosphates and phosphonates, the latter used as mimics of the phospholipid head groups. The bis-imidazolium hosts interacted with the guests in a cooperative manner leading to the presence of tight and well defined 1:2 ternary complexes. Optimized monomer combinations were subsequently used for imprinting of phosphatidic acid as an endotoxin dummy template. Presence of the aforementioned ternary complexes during polymerization resulted in imprinting of lipid dimers – the latter believed to crudely mimic the endotoxin Lipid A motif. The polymers were characterized with respect to template rebinding, binding affinity, capacity and common structural properties, leading to the identification of polymers which were thereafter subjected to an industrially validated endotoxin removal test. Two of the polymers were capable of removing endotoxin down to levels well below the accepted threshold (0.005 EU/mg API) in pharmaceutical production.

The biopharmaceutical industry is a multibillion-dollar industry which produces proteins and other drugs from microorganism fermentation^1^,^2^,^3^. Major side products from the fermentation processes are the endotoxins produced by Gram-negative bacteria, present in the fermentation broth and which can occur at any point within the process^4^. These toxins are thermally stable membrane-bound toxins that are released upon lysing of cells during the product workup. The endotoxins consist of a large group of compounds that contain a highly variable polysaccharide tail (containing the inner core, outer core and O-antigen) and a highly conserved lipid-A motif (Fig. 1A). The Lipid A motif consists of two glucosamine units, with a phosphate group on each carbohydrate and varying fatty acid chains on the rest of the molecule (Fig. 1B). Great efforts are currently employed in the removal process of traces of these toxins from the products, due to their severe immune response in humans: they cause endotoxemia, septic shock and possibly even death^5^. There is also a paramount need for Endotoxin sensors in order to replace the elaborate, unsustainable and expensive Limulus Amebocyte Lysate (LAL) method which has been raising environmental concerns due to the high mortality rate of horseshoe crab partly caused by the LAL industry.

To enable selective removal of a large group of endotoxins, the desired sorbent should focus on targeting the removal of these lipid-A motif phosphate groups. We have turned to molecular imprinting to generate such a sorbent^6^,^7^,^8^,^9^. Polymers are here synthesized in presence of a template which corresponds entirely or partly to the target that the polymers are designed to bind. Following the synthesis of the polymer, the template is removed, leaving behind a binding site complementary to the target molecule. Targets that are either scarce, unstable or toxic are not useful as templates and here we describe the rational behind the selection of the target substructure. Given the amphiphilic nature of the target, recognition driven by combined hydrophobic and polar interactions is expected to be particularly powerful^10^,^11^,^12^,^13^,^14^. Phosphatidic acid is a phosphomonoester and the common motif of the glycerophospho lipids. Being the most abundant structural lipid motif it is also widely commercially available in pure form. We reasoned that this lipid bears some structural resemblance with the Lipid A motif of Endotoxin by featuring a phosphomonoester and two fatty acid chains in a similar structural arrangement as in the toxin. Moreover, we anticipated that by promoting template dimerization the two phosphorylated glucosamines and four of the six fatty acid chains would be mimicked.

In order to specifically target the phosphate groups we turned to host-guest chemistry with its extensive literature on anion binding hosts. We have recently reported on a range of imprinted anion receptors for carboxylates^13^,^14^,^15^, phosphates^16^ and sulfates^17^ featuring 1,3-diaryl ureas as the common host monomer capable to engage as strong hydrogen bond donors with dioxyanion hydrogen bond acceptors (Fig. 2A). Moreover, such monomers proved useful for the imprinting of genotoxic arylsulfonates and the resulting scavengers were successfully applied in the degenotoxification of pharmaceuticals^18^.

In order to extend the solvent compatibility of such receptors to water and thereby be able to target endotoxin we decided to assess the use of monomers based on water compatible host motifs. Imidazolium derivatives as anion receptors have in this context been extensively investigated both in the form of low molecular weight hosts^19^,^20^ as well as in polymer bound form and in imprinting^21^,^22^,^23^. These powerful receptors act by engaging in strong [C–H]^+^ anion ionic hydrogen bonding interactions (Fig. 2B) which prevail in highly competitive media. Herein we have assessed such binding motifs in phosphate imprinting aiming at final anchoring of the lipid A backbone of endotoxin. We report on imprinted polymer design and characterisation and their final use for scavenging endotoxin using an industrial standard removal test.

## Results and Discussion

### Monomer solution interactions with phosphonate guest

**Figure 3:**
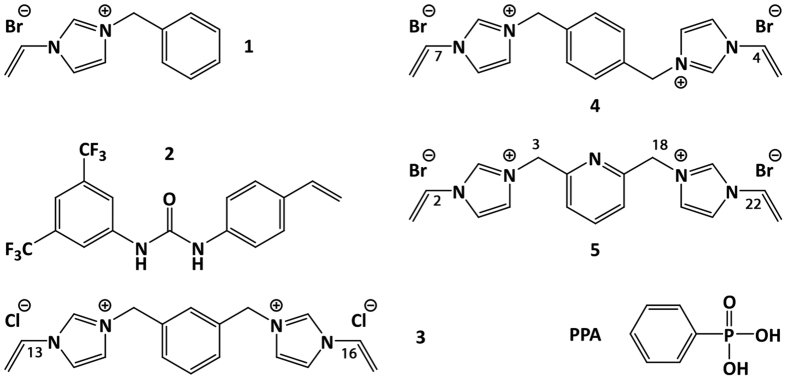
Selected monomers 1–5, for phosphate-selective MIPs and the template phenylphosphonic acid (PPA) used to probe monomer or polymer phosphate affinity.

Monomers 3 and 4 (Fig. 3) were readily prepared in one step from N-vinylimidazole and the corresponding halide whereas the synthesis of the remaining monomers was identical to those employed in previous reports^13^,^21^,^22^,^23^. Tetrabutylammonium-hydrogen-phenylphosphonic acid (PPA∙TBA) and bis-tetrabutylammonium- phenylphosphonate (PPA∙2TBA) were chosen as mono- and di-anion guests respectively, mimicking the phosphate groups of endotoxin.

The receptor monomer solutions (2 mM in d_4_-MeOH) were titrated with a standard solution of the anion guest up to a ten-fold molar excess. Table 1 shows the complexation induced shifts (CIS) of the protons used to calculate the given association constants (K_a_) and the complex stoichiometries determined by Jobs method of continuous variation (Supplementary Figures S1–S7)^24^.

The titration was accompanied by significant shifts of the host aryl and vinyl protons as indicated in Table 1 and those that could be monitored throughout the titration were used to calculate free concentrations and the binding curves in Supplementary Figures S1–S7. The imidazolium C-H protons were absent when performing the titrations in CD_3_OD indicating enhanced exchange of these protons or host deprotonation^25^. The imidazolium [C-H]^+^ hydrogen bond stabilization commonly inferred to play a role in aprotic solvents is less important in this solvent contrary to titrations performed in d6-DMSO where significant down-field shifts of these protons are seen.

Considering first interactions involving the monoureas and monoimidazoliums 1 and 2, no protons were shifted when titrating these monomers with PPA∙TBA showing that the interactions were very weak in this solvent. A different behavior was observed for the receptors 3–5 featuring two cationic sites and two hydrogen bond donor sites. Titrating 5 with PPA∙TBA (Table 1, entry 1) resulted in gradual downfield shifts of the host vinyl protons in accordance with a weak 1:1 host guest interaction. Since bis-imidazolium hosts typically show preference for binding dianions and multiply charged species^26^ we tested their ability to complex PPA∙2TBA. The dianion interacted strongly with 5 as viewed by the steep CIS plots (Supplementary Figure S2) and the sigmoidal shape of the curve indicated that the binding was cooperative^24^. Curve fitting using the Hill equation resulted in an association constant of K_a_ = 1680 M^−1^ and Hill slopes approaching 2 (Table 1, entry 2). This agreed with the Job’s plots in Supplementary Figure S2B supporting a preferred 1:2 host-guest stoichiometry. Such termolecular complexes has previously been observed for other bis-imidazolium hosts interacting with phosphate mono anions^26^,^27^. These have then been attributed to binding induced conformational changes^27^ in the host or has been left without explanation^26^. In the former case addition of excess guest was believed to promote a trans arrangement of the imidazolium groups allowing them to bind one guest molecule each. The guests were here far apart and it is unclear whether binding was cooperative in this case.

To shed light on the interaction mechanism we investigated the bis-imidazolium receptors 3 and 4 featuring the imidazolium groups bridged by meta or para substituted xylyl groups (Table 1, entries 3 and 4). Thus 3 corresponds to 5 but lacks the pyridine nitrogen. Interestingly, the latter contributes significantly to the affinity in view of the more than two fold lower K_a_ of 3 compared to 5. This heteroatom effect was also observed by Kim *et al*. comparing receptors comprising acridine versus antracene spacers, albeit targeting different guests – in this case phosphate monoanions. The effect was attributed to a additional hydrogen bond stabilization between the protonated guest and the heteroatom^26^. Given the absence of hydrogen bond donors in PPA∙TBA such stabilization is absent. It is therefore likely that the effect is due to steric factors.

As in the case of 5, both 3 and 4 bound their guests with strong cooperativity and with para-substituted 4 being the weaker binder. These results suggest that the imidazolium groups of hosts 3–5 act in concert to bind their guests by a multidentate mechanism rather than interacting independently with the guest. The cooperativity is likely due to coordination of both guest dianions to both imidazolium groups. Further studies are under way to elucidate the nature of these interactions.

Finally, we wanted to know to what extent the nature of the counter cation influenced the complex stability. Use of the sodium salts PPA·Na and PPA·2Na also led to the formation of 1:1 and 2:1 complexes with 5. In the latter case we observed an enhanced cooperativity and stability reflected in the high association constant (K_a_ = 2790 M^−1^) and a Hill slope of 3.5 (Table 1, entry 6). Interestingly, the use of a weak base to deprotonate PPA was detrimental to complex formation (Table 1, entry 7), presumably due to strong hydrogen bond competition from the protonated base.

### Preparation and characterisation of PPA imprinted polymers

**Figure 4:**
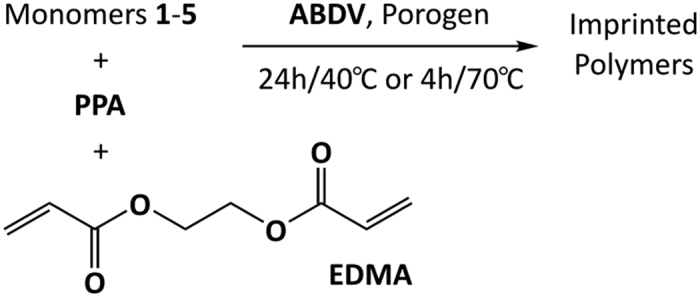
Procedure used for the synthesis of PPA imprinted polymers.

Imprinted polymers were prepared using these monomers and disodium phenylphosphonate as a divalent anion template to probe the general phosphate affinity of the imprinted hosts. The polymers were synthesized with either imidazolium (1, 3–5) or urea (2) based monomers as depicted in Fig. 4. The crosslinker selected was EGDMA used in a 40 to 1 ratio to the template.

**Figure 5:**
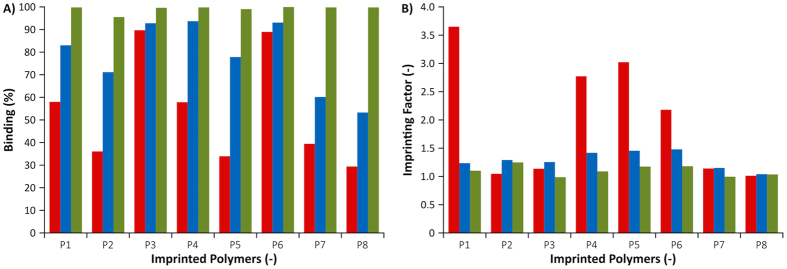
Uptake (% bound) (**A**) and imprinting factor (**B**) of PPA·2Na or PPA·Na (P2) on PPA imprinted polymers after incubation in HEPES buffer (0.01 M, pH 7)/MeCN:90/10 (red bars), MeOH (blue bars) or MeCN (0.1% PMP) (green bars).

The formulation of each polymer is described in Table 2 and the preparation procedure reported in the Experimental Section. Removal of template was investigated by HPLC concluding that at least 85% was removed by the solvent extraction. The amount of bound PPA to the imprinted polymers in three different solvent systems and the enhanced binding due to imprinting was then investigated and the results are shown in Fig. 5.

Considering first the results from the binding experiment in MeCN (0.1% PMP), it is seen that PPA binds strongly to all polymers and is completely depleted in all solutions. Hence no ranking of the polymers performance can be done based on these results. Binding is weaker in methanol and even more so when incubating in an aqueous buffer (HEPES, pH7). In the latter two cases, the general ranking of the polymers in terms of PPA uptake is essentially the same. The binding results in HEPES buffer will be discussed below. The lower uptake to P2 compared to P1 is the simple consequence of the former featuring half the number of cationic imidazolium groups per unit weight of polymer. Hence, this should reflect the importance of the imidazolium group in the general binding mechanism of the oxyanion guest. A polymer prepared using the neutral urea-based monomer 2 as a second functional monomer in addition to the cationic 1 (P3 versus P1), results in a large increase in PPA uptake from 58% to 90% but no increase in the imprinting factor.

This appears to be a cooperative effect given the weak binding and essentially absence of imprinting for the polymers prepared using the urea monomer alone (P7, P8). Moving to the polymers (P4–P6) prepared from the bis-imidazolium host monomers (3–5) the binding of PPA is accompanied by imprinting factors >1 and is thus clearly related to imprinting. Binding of PPA to these polymers scales with the relative ranking of the host monomers with respect to their association constants with PPA (Table 1). Among the three host monomers, weakest binding is thus seen using the para-substituted monomer 4 where the placement of the cationic and hydrogen bond CH donors is poorly set up for cooperative binding to a phosphate anion. The polymer prepared using the better complement, the meta-substituted monomer 3, (P4) features a stronger affinity for PPA (58%) whereas P6 prepared using the meta-substituted monomer 5 with the pyridine spacer shows the highest affinity for PPA (89%). Monomer 5 was therefore used for the synthesis of polymer complements to endotoxin using the PA template.

### Preparation and characterisation of PA imprinted polymers

**Figure 6:**
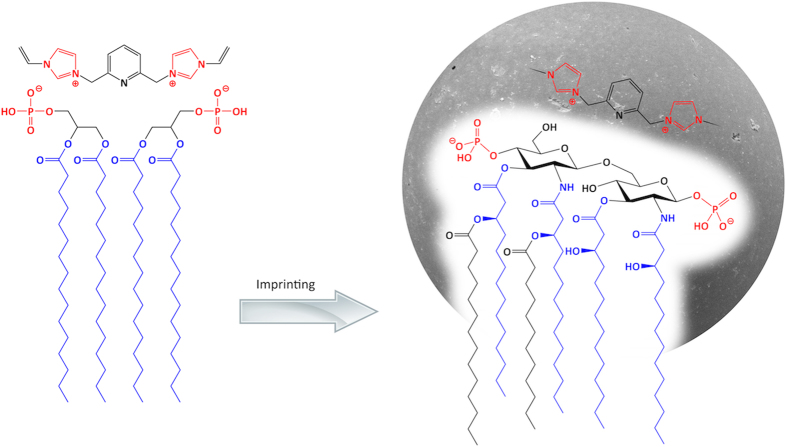
Hypothetical structure of a polymer complement to Lipid A by imprinting of a PA dimer.

In order to obtain a polymer complement to endotoxin we aimed at imprinting phosphatidic acid (PA) in the dimeric form as depicted in Fig. 6. The strong tendency of 5 to coordinate two guest species and a hydrophobic stabilization due to the lipid chains we anticipated would favor such an arrangement.

PA-imprinted polymers were prepared as described in the experimental section using host monomer 5 and PA•Na and different template-monomer stoichiometries (see Table 2).

To exploit the cooperative effects observed in the PPA imprinting (Fig. 5) we assessed also the addition of 2 as a second functional monomer. TGA and ^31^P NMR were employed to investigate the removal of the template. Solid state ^31^P NMR before and after soxhlet extraction (Supplementary Figure S9) showed a large decrease of the phosphorous signal confirming a near quantitative template removal. Nevertheless there was still a weak phosphorus signal which indicates that the removal was incomplete. The differences in decomposition profiles before and after Soxhlet extractions were used for elucidation of the degree of template removal^28^. The decomposition profile of PA (Supplementary Figure S10) showed that the major decomposition of the template was the loss of fatty acids and glycerol by ~400 °C, whereas ~15% of the molecule corresponding to the inorganic NaPO_3_ part remained up to 1000 °C. The TGAs of a PA imprinted and a nonimprinted polymer carried out before and after Soxhlet extraction (Supplementary Figure S11) showed that the extracted polymers decomposed nearly quantitatively leaving less than 1% final residue. On the other hand the polymer that still contained template had significantly higher residue after decomposition of the organic parts. After the Soxhlet extraction decrease in polymer residual suggests that the majority of the template was removed (ca 90%).

The amount of bound PA to the imprinted polymers in different solvent systems and the enhanced binding due to imprinting was then investigated by different techniques. The three quantification procedures that were tested were ^31^P NMR based on internal reference, iron(III)-salicylate (Fe(III)Sal_3_) colorimetry and turbidity measurements^29^. The ^31^P NMR method was due to its low sensitivity only useful for measuring high concentrations of the template (Supplementary Figure S12). Due to the ^31^P 100% abundance and the absence of signals originating from other phosphorus compounds the ratio of Ph_4_PCl to the template could be relatively easily determined. The Ph_4_PCl to template ratio showed a linear correlation with respect to the concentration of template but the technique is restricted to a limited concentration interval due to solubility issues. For the intermediate concentration (3 mg/mL) the peak integrals revealed that ca 50% of PA added was bound to the MIP whereas the NIP showed no binding of template.

**Figure 7:**
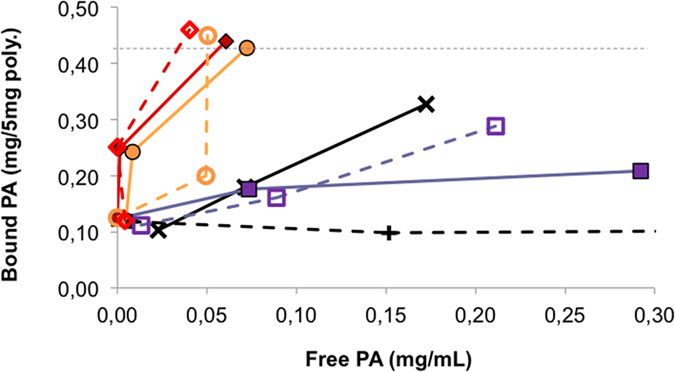
Binding curves showing binding of PA to imprinted (solid lines) and nonimprinted (dashed lines) P9 (black crosses); P10 (violet squares); P11 (orange circles) and P12 (red diamonds) as a function of the concentration of free PA in ammonium bicarbonate buffer (5 mM, pH 6.5). The UV absorbance was measured at 490 nm, normalized to 0 mg/mL with respect to template leaching. The standard deviations for each measurement was SD < ±0.03 mg/mL. The dashed line indicates the nominal capacity of the materials assuming quantitative template incorporation.

The use of other spectroscopic techniques, such as iron(III)-salicylate colorimetry, was useful for determining binding at lower concentrations of template and in other solvent systems. The Fe(III)Sal_3_ colorimetric assay measured the discoloration of the Fe(III)Sal_3_ complex by ligand displacement induced by phosphate (Supplementary Figure S13) and the assays could hence be performed by parallel analysis using a UV plate reader. Solutions of PA (0–1 mg/mL) were incubated at 50 °C for 48 h with the polymers P9–P12, and subsequently the reduction in template concentration in the supernatant was determined. We first investigated the template rebinding in NH_4_HCO_3_ buffer (Fig. 7).

The phospholipid was soluble only at high temperatures and started to precipitate at room temperature. Due to this fact and the limited sensitivity of the assay the concentration interval was restricted to 0.125–0.5 mg/mL. In spite of this limitation, conclusions concerning the relative binding affinity and selectivity could be drawn. Polymers P9 and P10 displayed shallow binding curves indicating a relatively weak affinity. Much stronger binding was displayed by the urea containing P11 and P12 resulting in a nearly complete depletion of PA from the supernatant. In agreement with the results discussed for PPA, introduction of the urea monomer 2 strongly favors binding of the phosphate. Especially interesting is the strong binding observed on P12 which is produced using a 2/1 template/host monomer ratio. Assuming that this reflects the presence of sites templated against the PA dimer as depicted in Fig. 6 this polymer should offer a better complement to the endotoxin target.

### Binding of endotoxin to the imprinted polymers

**Figure 8:**
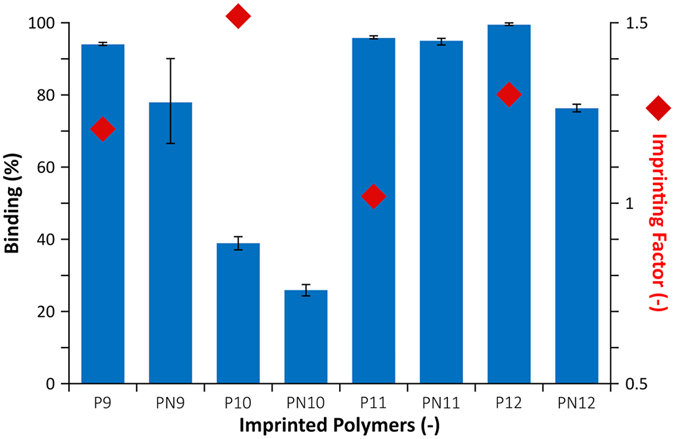
Uptake (% binding: blue bars) and imprinting factor (red dots) of endotoxin on PA imprinted polymers determined by the LAL assay.

Endotoxin scavenging was successfully assessed featuring endotoxin standards. Due to the complexity of handling and analyzing Endotoxins, the most promising polymers, namely P9–P12 (Table 2), were selected for the microbiology tests. The binding studies were carried out in aqueous solutions with initial endotoxin concentration of 10 EU/mL (EU = endotoxin unit). The final concentrations were determined by Limulus amebocyte lysate (LAL) assay after 24 h incubation with 5 mg of the polymers (Fig. 8).

The strongest binding was observed with polymers P11 and P12 which had the mix of the imidazolium monomer 5 and urea monomer 2. This agrees with the binding results of the PA standard in Fig. 8 and suggests that the imidazolium monomer 5 combined with urea monomer 2 leads to particularly high target affinity. Also in line with the standard PPA and PA binding tests above is the lower binding affinity of P10, prepared using urea monomer 2, compared to P9 containing only imidazolium monomer 5 as functional monomers. Furthermore, all polymers except P11 display significant imprinting as shown by IF values exceeding 1. Interestingly, the highest uptake and imprinting factor was recorded for polymer P12, prepared from a 2/1 PA/monomer ratio. This possibly reflects the anticipated stabilization of PA dimers prior to polymerization which in turn creates a better complement to endotoxin. The threshold limit set by regulatory agencies for endotoxin levels in active pharmaceutical ingredient (API) is 0.005 EU/mg API. A typical industrial scenario with a concentrated 100 mg/mL API stream requires the use of 33 g P12/kg API.

## Conclusion

We have shown that polymerizable bis-imidazolium hosts can effectively coordinate phosphate and phosphonate guests in 1:2 host guest stoichiometry. This leads to well defined ternary complexes allowing imprinting of template dimers. Imprinting of such motifs are of significant interest when the goal is to capture multimeric species^30^,^31^ or as in this case for creating binding sites complementary to complex lipids using simple affordable lipids as templates. The resulting receptors were successfully tested for endotoxin removal using an industrially approved endotoxin assay. 33 g of scavenger per kg of product is needed to achieve industrially acceptable API purity. Significant binding of endotoxin was observed, and the highest binding and imprinting factor was attained for polymer P11 and P12 with a ratio of template: monomer 5: monomer 2 equal to 1:1:1 and 2:1:1 respectively. These results were consistent with the results from PA binding tests and suggests an applicability of the scavengers for endotoxin removal from active pharmaceutical ingredients (API). The performance requirements in this context is defined by the Endotoxin threshold and the API concentration. Assuming 0.005 EU/mg-API as the Endotoxin threshold and an API concentration of 100 mg/mL, the scavenger needs to be able to remove endotoxin down to a concentration of 0.5 EU/mL. This criteria is met by P11 (0.4863 EU/mL) and P12 (0.1117 EU/mL) at 33.3 g-MIP/kg-API. Hence, we have demonstrated that lipid imprinted polymers can offer a realistic alternative for scavenging of endotoxins in pharmaceutical production.

## Methods

### Preparation of phenylphosphonate imprinted polymers

The composition of each polymer is given in Table 2. The following general procedure was used for preparing polymers P1–P6. Disodium phenylphosphonate (PPA•2Na) or monosodium phenylphosponate (PPA•Na) (P2 only) (0.125 mmol), functional monomer, EGDMA (5 mmol) were dissolved in methanol (1.4 mL). The initiator ABDV (0.05 mmol) was added to the solution. The solution was transferred to a glass ampoule, cooled to 0 °C, and purged with a flow of dry nitrogen for 15 min. The tubes were then flame-sealed while still cooling, and the polymerization initiated by placing the tubes in a water bath heated to 40 °C. The tubes were left in the bath for 24 h and then transferred to an oven and kept there at 70 °C for 4 hours. The tubes were broken after 24 h and the polymers lightly crushed. They were washed thereafter with 3 × 10 mL MeOH/0.1 M HCl (1:1), and the wash fractions analyzed by LCMS. Non-imprinted polymers (PN1–PN6) were prepared in the same manner described above, but with the omission of the template molecule from the prepolymerization solution.

### Preparation of phosphatidic acid imprinted polymers

The compositions of the phosphatidic acid (PA) imprinted polymers P9–P12 are given in Table 2. The polymers were prepared following a similar procedure as for the PPA imprinted polymers however starting from the monosodium salt of phosphatidic acid (PA•Na) and methanol/toluene: 1/1 as porogen. The polymers were lightly crushed and extracted in a Soxhlet apparatus with methanol. The polymers were then further crushed and sieved to 25–50 μm and sub-25 μm particles. These particles were used as solid phases for extraction and binding tests.

### ^1^H NMR spectroscopic titrations and estimation of binding affinities and stoichiometries

The complex stoichiometry was first assessed using the Job method of continuous variation. Stock solutions of the host monomer and guest (10 mM and 25 mM in DMSO-d6 or methanol-d4, respectively) were combined in NMR tubes, thereby resulting in the following molar ratios: 0:10, 2:8, 3:7, 4:6, 5:5, 6:4, 7:3, 8:2, 10:0. Total concentration of host and guest was 2 mM. ^1^H NMR spectra were thereafter recorded and the proton signals, which could be monitored for all apart from the 0:10 mixing ratios, were used for the evaluation of the complex stoichiometry. ^1^H NMR spectroscopic titrations were performed in dry deuterated solvents. The dissociation (K_d_) association constants (K_a_) for the interaction between the hosts and guests were determined by titrating an increasing amount of guest (PPA∙TBA, PPA∙2TBA, PPA·Na or PPA∙2Na) into a constant amount of functional monomer (1–5). The concentration of the functional monomer was 2 mM and the amount of added guest was 0, 0.25, 0.5, 0.75, 1.0, 1.5, 2.0, 4.0, 6.0, and 10.0 equivalents. The complexation induced shifts (CISs) of relevant protons were followed and titration curves were constructed of CIS versus free guest concentration (c). The raw titration data were fitted to a 1:1 binding site model (equation 1),


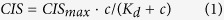


where *CIS*_*max*_ is the maximum CIS at saturation and *K*_*d*_ is the dissociation constant, or to a cooperative binding site model (equation 2):


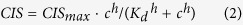


where *h* is the Hill slope^24^. The fitting was performed by nonlinear regression using GraphPad Prism 7 (GraphPad Software, La Jolla, CA, USA), from which the dissociation constants were calculated. The association constant K_a_ was calculated as the inverse of the dissocation constant.

### Batch binding tests of PPA imprinted polymers

Binding tests were performed in order to probe the particles affinity for PPA. Dry template free particles (10 mg) were suspended in 1 mL of 1 mM solutions of PPA•2Na (PPA•Na for P2) in 1.5 mL microfuge tubes. After a 24 h incubation at room temperature by gentle shaking the solutions were filtered and transferred into HPLC vial inserts and were analyzed by reversed phase HPLC analysis, using a polar endcapped C18 reverse phase Luna (250 × 4.6). The mobile phase consisted a mixture of methanol and water (0.1% of TFA) 32/68 for PPA at flow rate of 1 mL/min. The injection volume was 5 μL. The column was kept at room temperature. The absorbance wavelength was 205 nm for PPA. For quantification purposes calibration standards were prepared in the same conditions that the rebinding experiments were made. The calibration curve was prepared in 0.1–1.2 mM concentration range with R^2^ = 0.9994. Retention time of PPA is 5.7 min. The specific amount of solute bound by the polymeric particles (B) was determined by the following formula:


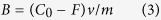


where *C*_0_ is the initial solute concentration, *F* is the final solute concentration in the supernatant, *v* (mL) is the total volume of the adsorption mixture, and *m* is the mass of polymer in each vial. The imprinting factor (IF) was calculated as the ratio of bound solute on an imprinted polymer (MIP) over that bound on a corresponding nonimprinted polymer (NIP) as:


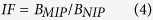


### Batch binding tests and adsorption isotherms of PA imprinted polymers

The rebinding experiments performed on the PA imprinted polymers (P9–P12) were conducted by different techniques. The tests were carried out using 5 mg of the sub-25 μm polymer particle fraction which were added to a known concentration of standard solutions of PA•Na in 1 mL of rebinding solvent, and were allowed to incubate for 48 h at room temperature with rolling agitation. Subsequently the solutions were centrifuged for 1–5 min to sediment the particles. The liquid was collected and the remaining concentration was determined. The three quantification procedures that were tested were based on (i) ^31^P NMR experiments in the presence of an internal reference, (ii) iron(III)-salicylate (Fe(III)Sal_3_) colorimetry^29^ and (iii) turbidity measurements.

The specific amount of solute bound by the polymeric particles (B) was determined as previously described for the PPA binding tests. Binding curves were constructed by plotting B against free concentration F.

### Endotoxin removal test

Limulus Amebocyte Lysate (LAL) is an *in vitro* endotoxin test for human and animal parenteral drugs. LAL is an enzyme, extracted from the blood of the horseshoe crab. The standard Endotoxin stock solution is prepared from the Endotoxin reference standard which has been assayed against the WHO international standard for Endotoxin. The Endotoxin content is expressed in the international Endotoxin Unit (EU). The 1000 EU/mL concentrated stock solutions of Endotoxin was prepared by rehydrating the lyophilized endotoxin standard in 5 mL LAL reagent water. The 10 EU/mL diluted stock solution was prepared by diluting 300 μL of the 1000 EU/mL concentrated stock solution to 30 mL with LAL reagent water. The diluted stock solution was subsequently used as the loading solution in the bulk rebinding MIP studies. The FDA approved and validated kinetic chromogenic LAL method was applied to quantify the endotoxin content before and after the application of MIPs. A 5 point calibration was inferred for the Endotoxin quantification ranging from 0.005 to 50 EU/mL resulting in 0.005 EU/mL LOQ and 0.001 EU/mL LOD. The analysis of each sample was carried out twice and the mean value is herein presented. Prior to analysis the supernatants were filtered using a 5 μm Millipore filter. All the samples, standards and reagents were stored at 6 °C. For safety precautions and in order to avoid contamination of the samples with endotoxins, all of the equipment used in the study were depyrogenated at 250 °C or purchased as depyrogenated material. The LAL analyses were carried out on Sunrise Tecon plate reader, and the control of the system was carried out on EndoScan-V version 3.1.4 acquisition software including data collection and processing.

Prior to the Endotoxin bulk rebinding tests, the scavengers were subjected to mild depyrogenation by extensive washing with 0.1 M aq. HCl solution followed by washing with LAL reagent water. 5 mg of depyrogenated P9, P10, P11 and P12 were placed in depyrogenated vials and 1.5 mL 10 EU/mL diluted Endotoxin stock solution was loaded on the scavengers. The rebinding mixtures were shaken for 24 hours, and the supernatants were filtered and subjected to LAL testing.

## Additional Information

**How to cite this article:** Sulc, R. *et al*. Phospholipid imprinted polymers as selective endotoxin scavengers. *Sci. Rep.* **7**, 44299; doi: 10.1038/srep44299 (2017).

**Publisher's note:** Springer Nature remains neutral with regard to jurisdictional claims in published maps and institutional affiliations.

## Supplementary Material

Supplementary Information

## Figures and Tables

**Figure 1:**
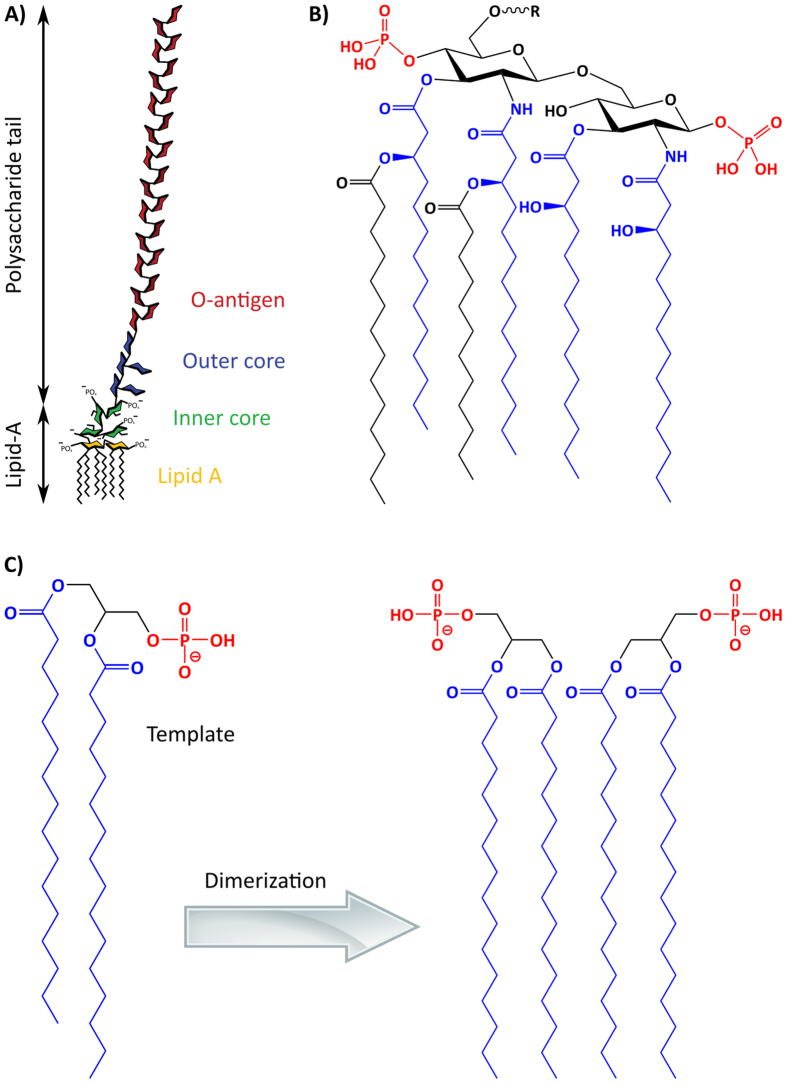
Endotoxins. (**A**) Generic layout of endotoxins. (**B**) Highly conserved Lipid A motif of endotoxins (R at the top of molecule represents the polysaccharide tail). (**C**) Phospholipid template, which in the form of dimers mimic four of the six fatty acids of endotoxin.

**Figure 2:**
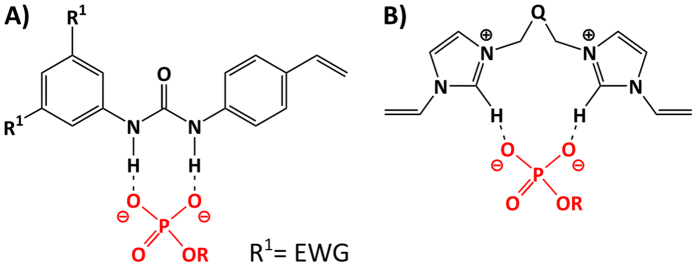
Monomers and binding ability. Theoretical hydrogen bonding interactions with the target phosphate group and (**A**) 1,3-diarylurea host monomers or (**B**) bis-imidazolium host monomers.

**Table 1:** Association constants, stoichiometries and complexation induced shifts for complexes formed between bis-imidazolium host monomers 3–5 and phenylphosphonic acid (PPA) guests in methanol-d4.

Entry	Host monomer^b^	Guest	Proton^b^	K_a_ (M^−1^)^a^	Cplx (H:G)	CIS^a^ (ppm)	h^a^
1	5	PPA∙TBA	CH (2, 22)	n/a	1:1	0.015	n/a
2	5	PPA∙2TBA	CH (2, 22)	1680 ± 121	1:2	0.15	1.9
3	3	PPA∙2TBA	CH (13, 16)	751 ± 62	1:2	0.09	2.2
4	4	PPA∙2TBA	CH (7, 4)	515 ± 41	1:2	0.09	2.2
5	5	PPA∙Na	CH (2, 22)	21 ± 8	1:1	−0.03	1.1
6	5	PPA∙2Na	CH (2, 22)	2790 ± 263	1:2	0.10	3.5
7	5	PPA∙2PMP	CH (3, 18)	59 ± 5	1:1	0.09	1.0

^a^Average association constants (K_a_), complexation induced shifts (CIS) and Hill slopes (h) based on the shift values of the resonance signals indicated.

^b^Monomers and assignements according to the numbering shown in Fig. 3.

**Table 2:** Composition of imprinted polymers.

Polymer	Template^b^ (T)	Funct. mon. 1 (FM1)^c^	Funct. mon 2 (FM2)^c^	Molar ratio T/FM1/FM2/EGDMA	Solvent
P1	PPA2·Na	1	—	1/2/0/40	MeOH
P2	PPA·Na	1	—	1/1/0/40	MeOH
P3	PPA·2Na	1	2	1/2/2/40	MeOH
P4	PPA·2Na	3	—	1/1/0/40	MeOH
P5	PPA·2Na	4	—	1/1/0/40	MeOH
P6	PPA·2Na	5	—	1/1/0/40	MeOH
P7^a^	PPA	2	—	1/1/0/40	THF
P8^a^	PPA	2	—	1/2/0/40	THF
P9	PA·Na	5	—	1/1/0/40	MeOH/Tol
P10	PA·Na	2	—	1/1/0/20	MeOH/Tol
P11	PA·Na	5	2	1/1/1/40	MeOH/Tol
P12	PA·Na	5	2	1/0.5/0.5/40	MeOH/Tol

^a^The procedure used for preparing polymer P7 and P8 was the same as we have reported previously with a molar ratio PPA/PMP/monomer 2/EGDMA of 1/1/1/40 using THF as porogen^16^.

^b^The degree of template removal for polymers P1–P6 was: 89, 84, 85, 87, 93 and 88% respectively.

^c^Functional monomers used according to the numbering shown in Fig. 3.
