# Supplementary material for: Phospholipid imprinted polymers as selective endotoxin scavengers
Source: Sci Rep. 2017 Mar 17;7:44299. doi: 10.1038/srep44299 (PMC5358689; doi:10.1038/srep44299)
Supplement: Supplementary Information [file srep44299-s1.pdf]

# Supplementary Information

# Phospholipid imprinted polymers as selective endotoxin scavengers

Robert Sulc, Gyorgy Szekely, Sudhirkumar Shinde, Celina Wierzbicka, Filipe Vilela, David Bauer and Börje Sellergren*

## Table of Contents

Experimental ........ S2

Figure S1. $^{1}H$ NMR titration curves showing the complexation induced shifts (CIS) of diagnostic protons of 5 upon increasing additions of PPA·TBA........ S6

Figure S2. $^{1}H$ NMR titration curves showing the complexation induced shifts (CIS) of diagnostic protons of 5 upon increasing additions of PPA·2TBA........ S7

Figure S3. $^{1}H$ NMR titration curves showing the complexation induced shifts (CIS) of diagnostic protons of 3 upon increasing additions of PPA·2TBA........ S8

Figure S4. $^{1}H$ NMR titration curves showing the complexation induced shifts (CIS) of diagnostic protons of 4 upon increasing additions of PPA·2TBA........ S9

Figure S5. $^{1}H$ NMR titration curves showing the complexation induced shifts (CIS) of diagnostic protons of 5 upon increasing additions of PPA·Na ........ S10

Figure S6. $^{1}H$ NMR titration curves showing the complexation induced shifts (CIS) of diagnostic protons of 5 upon increasing additions of PPA·2Na ........ S11

Figure S7. $^{1}H$ NMR titration curves showing the complexation induced shifts (CIS) of diagnostic protons of 5 upon increasing additions of PPA·2PMP ........ S12

Figure S8. $^{1}H$ NMR spectra of 5 ........ S13

Figure S9. Solid state $^{31}P$ NMR of a PA imprinted polymer ........ S14

Figure S10. a) Molecular weights of fragments of PA. b) Thermal gravimetric analysis (TGA) of PA. ........ S15

Figure S11. TGA of a PA imprinted polymer . ........ S16

Figure S12. Comparison of $^{31}P$ NMR of a PA standard 3mg/mL and the supernatant isolated after incubation with a PA imprinted polymer. ........ S17

Figure S13. The Fe(III)$Sal_3$ colorimetric phosphate assay ........ S17

## Experimental

Ethylene glycol dimethacrylate (EGDMA), tetrabutylammonium hydroxide (TBAOH; 1 M in methanol), methanol (MeOH), dry dichloromethane (DCM), magnesium sulfate and 1-vinylimidazole were purchased from Sigma-Aldrich (Steinheim, Germany). DMSO-d6, methanol-d4 ($CD_3OD$), sodium hydrogen carbonate ($NaHCO_3$), acetone and ethyl acetate were obtained from VWR (Radnor, PA, USA). Other solvents were of reagent grade or higher. 1,3-bis(bromomethyl) benzene; vinyl imidazol; 2,6-bis-(bromomethyl)pyridine and pentamethylpiperidine (PMP) were purchased from Sigma Aldrich (Steinheim, Germany) and bromomethyl benzene, 1,4-bis(chloromethyl) benzene from Across. Dry acetonitrile was purchased from Merck (Darmstadt, Germany). *N*,*N*'-Azo-bis-(2,4-dimethyl)valeronitrile (ABDV) was purchased from Wako Chemicals GmbH (Neuss, Germany. DMSO-*d*6 was purchased from Deuterio-GmbH (Kastellaun,Germany).

The templates Phenylphosphonic acid (PPA), its sodium salts (PPA•Na, PPA•2Na), Phosphatidic acid • Na (PA•Na: 1,2-Dipalmitoyl-sn-glycero-3-phosphate monosodium salt) were purchased from Sigma Aldrich (Steinheim, Germany).

Ethylene glycol dimethacrylate (EGDMA) was purified by the following procedure prior to use: the received material was washed consecutively with 10% aqueous NaOH, water and brine. After drying over MgSO4, pure and dry EGDMA was obtained by distillation under reduced pressure. All other reagents were used as received.

The functional monomers 3-benzyl-1-vinyl-1*H*-imidazolium bromide (1),[21] *N*-3,5-bis(trifluoromethyl)-phenyl-*N′*-4-vinylphenylurea (2),[13] and 1,1'-[2,6-pyridindiylbis(methylene)]bis[3-vinyl]-1*H*-imidazolium dibromide (5),[22] were synthesized as reported before. E.coli Control Standard Endotoxin, Lyophilized Endosafe Endochrome-K reagent and LAL reagent water (<0.001 EU/mL) were purchased from Charles River Endosafe division of Charles River Laboratories, Inc (Charleston, SC, USA). Hydrochloric acid (HCl) was purchased from Fluka (Germany).

**Apparatus and methods**

$^{1}$H NMR spectra were recorded on a Bruker Omega 500 (500 MHz) and on a Varian Mercury 400 MHz instrument. The chemical shifts were reported in ppm using deuterated solvent peaks as the internal references. Coupling constants (*J*) were reported in Hz and the splitting abbreviations used are: s, single; d, doublet; t, triplet; q, quartet; sep, septet; m, multiplet; br, broad. $^{13}$C NMR spectra were obtained using the same instruments operating at 125.8 MHz. Solid $^{31}$P NMR spectra were obtained using Bruker Omega 500 for solid NMR operating at 161.99 MHz. Solution $^{31}$P NMR spectra were obtained using Bruker for solid NMR operating at 121.50 MHz with inverse gated decoupling.

Carbon, nitrogen, and sulfur contents were determined by elemental analysis at the Department of Organic Chemistry, Johannes Guttenberg Universität Mainz using a Heraeus CHN-rapid analyzer (Hanau, Germany).

High Performance Liquid Chromatography (HPLC). The HPLC measurements were carried out on Hewlett-Packard HP 1050 instruments (Agilent Technologie, Waldbronn, Germany) equipped with an autosampler and a UV diod array detector.

Thermogravimetric Analysis (TGA) was carried out using a TGAQ50 (TA Instruments, ESchborn, Germany). The sample (10–15 mg) was placed in a platinum pan, which was suspended in a sensitive balance together with the reference pan. The sample was then heated in a furnace with a heating rate of 20 °C/min, under $N_2$ atmosphere.

The concentration of PA in microtiterplate scale was determined as follows. 100 µl of FeSal solution (2 mM $FeCl_3$, 12 mM sodium salicylate, 1 M HCOOH/NaOH, pH 4.0) were added to the samples containing unknown amounts of PA (80 µl). After equilibriation for 5 min at room temperature, the absorbance of the samples was measured at 490 nm yielding the PA concentration by means of a calibration curve obtained using PA standards in the same medium.

**Synthesis of mono and bis-imidazolium based monomers**

To prevent polymerization, a spatula tip of sulphur was added to reaction mixture. The general workup was performed after overnight refluxing. After cooling to room temperature, the solvent was evaporated to dryness under vacuum. The resulting solid was then redissolved in ethanol and precipitated out with the addition of diethyl ether, to afford the desired solid product.

*1-Vinyl-3-{3-[(1-vinyl-1H-imidazol-3-ium-3-yl)methyl]benzyl}-1H-imidazol-3-ium dibromide (3)*

A solution of 1,3-bis(bromomethyl)benzene (Aldrich, 2g, 3.78mmol) and vinylimidazole (Aldrich, 1.4ml, 7.57mmol) in acetonitrile (Merck, 60ml) was refluxed over night and otherwise following the general protocol. Yield: 72%, $^{1}$H NMR (400 MHz, DMSO) δ 5.42-5.45 (m, 2H), 5.43, 5.45, 5.45, 5.52 (s, 4H), 5.99-6.04 (m, 2H), 7.33-7.39 (m, 2H), 7.50 (s, 3H), 7.68 (s, 1H), 8.01(s, 2H), 8.29 (s, 2H), 9.80 (S, 2H); $^{13}$C NMR (101 MHz, DMSO) δ 51.84, 108.89, 119.45, 123.31, 128.80, 128.86, 128.96, 129.70, 135.10, 135.62. Calculated C 47,81%, H 4,46% , N 12,39%; found C 46,08%, H 4,96 %, N 12.28%. MS (ESI+) 291,08788; 373,8568.

*1-Vinyl-3-{4-[(1-vinyl-1H-imidazol-3-ium-3-yl)methyl]benzyl}-1H-imidazol-3-ium dichloride (4)*

A solution of 1,4-bis(chloromethyl)benzene (Acros, 0.86, 5mmol) and vinylimidazole (Aldrich, 0.45ml, 5mmol) in acetonitrile (Merck, 25ml) was refluxed over night and otherwise following the general protocol. Yield: 40%; $^{1}$H NMR (300 MHz, DMSO) δ 5.39 - 5.42 (d, 2H), 5.53 (s, 4H), 6.00-6.06 (m, 2H), 7.33-7.42 (m, 2H), 7.58 (s, 4H), 8.04 (s, 2H), 8.31 (s, 2H), 10.03 (s, 2H); $^{13}$C NMR (75 MHz, DMSO) δ 51.65, 108.78, 119.50, 123.27, 128.92, 129.26, 135.11, 135.73. Calculated C 59, 51%, H 5.55 % , N 15,42%; found C 53,27%, H 6,65 %, N 13,82%. MS (ESI+) 291,0031; 328,9725.

A.

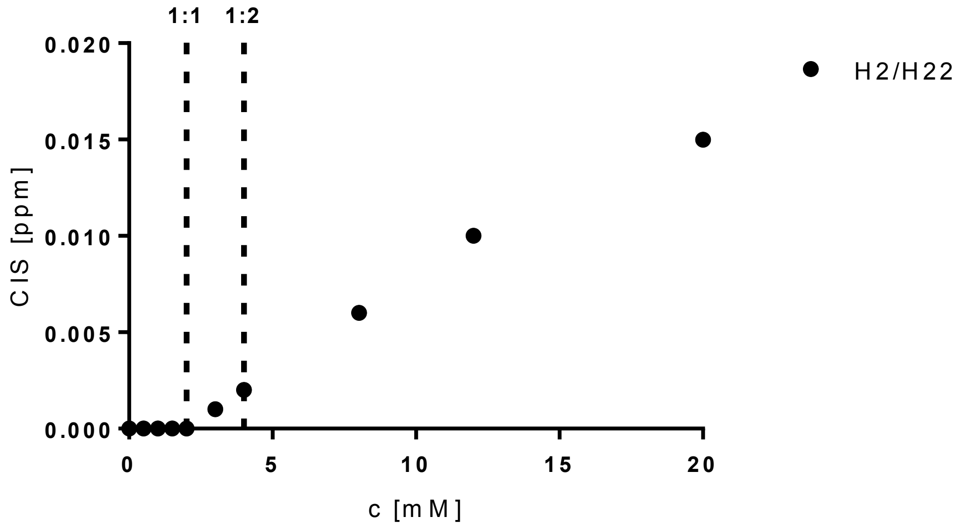


B.

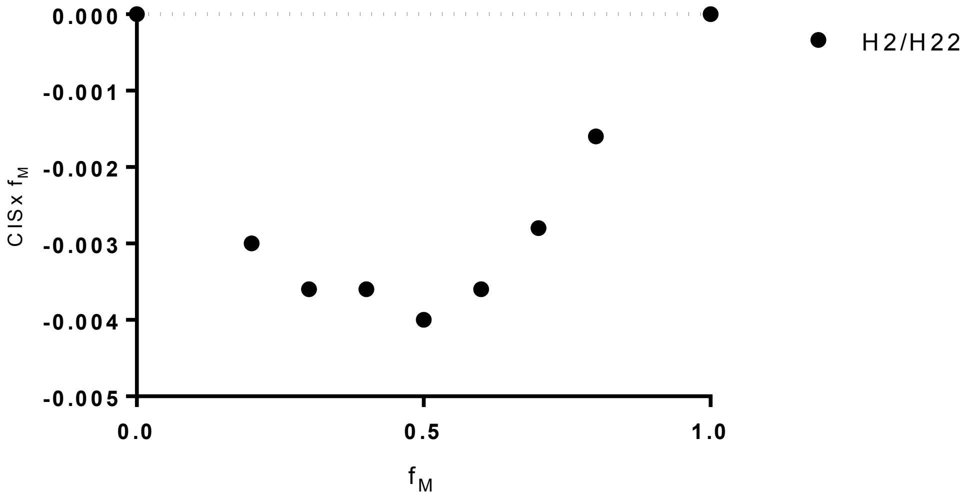


Figure S1. (A) $^{1}$H NMR titration curves showing the complexation induced shifts (CIS) of diagnostic protons of 5 upon increasing additions of PPA·TBA in $CD_3OD$. c=concentration of free guest. The dashed lines represent 1 and 2 equivalents of added guest with respect to host. (B) Job's plot corresponding to the system in A.

A.

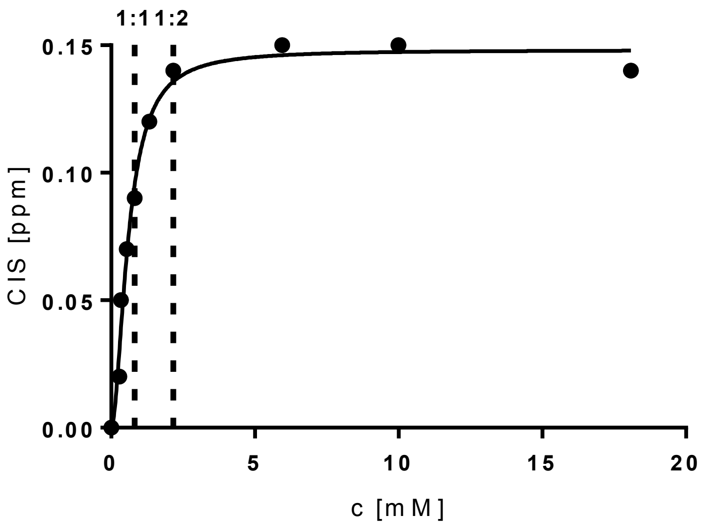


B.

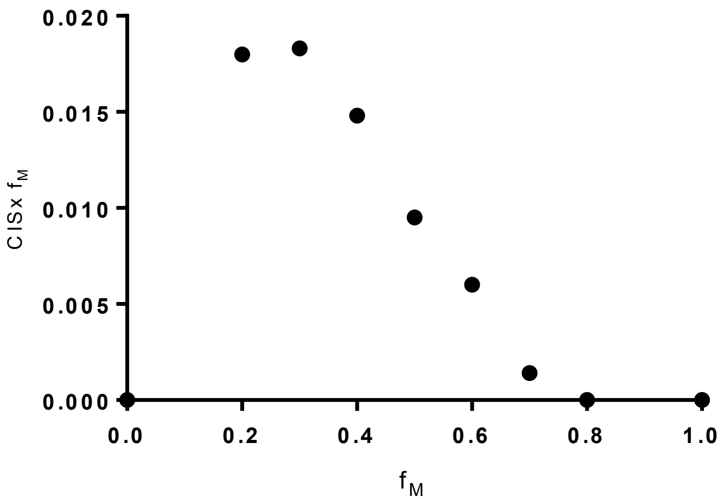


Figure S2. (A) $^{1}H$ NMR titration curves showing the complexation induced shifts (CIS) of diagnostic protons of 5 upon increasing additions of PPA·2TBA in $CD_3OD$ c=concentration of free guest. The dashed lines represent 1 and 2 equivalents of added guest with respect to host. (B) Job's plot corresponding to the system in A.

A.

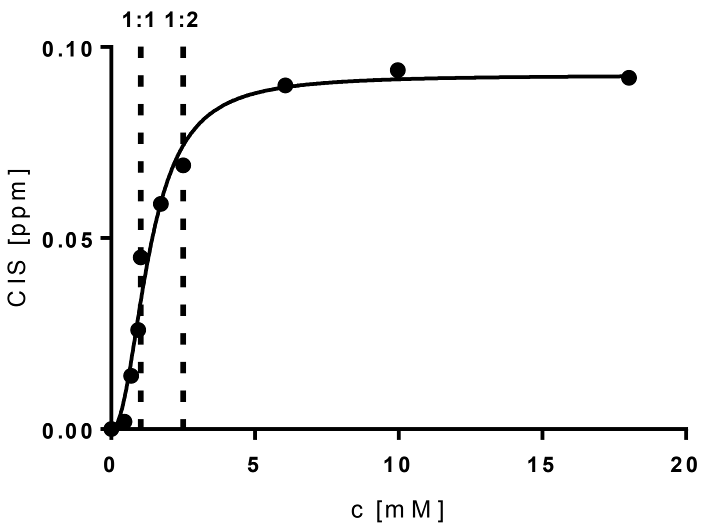


B.

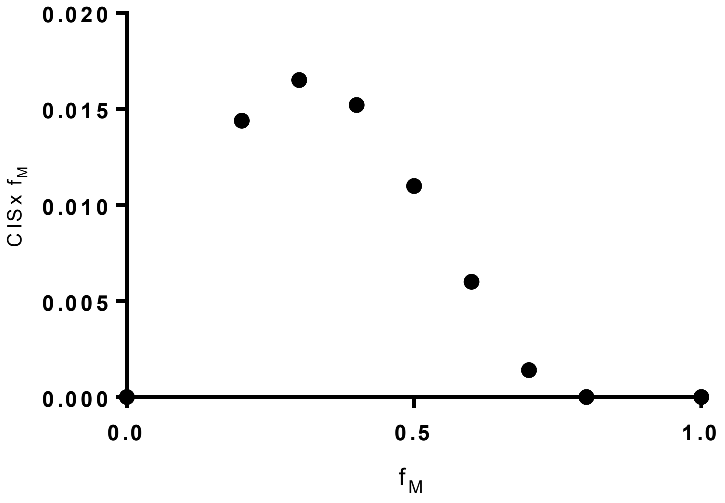


Figure S3. (A) $^{1}H$ NMR titration curves showing the complexation induced shifts (CIS) of diagnostic protons of 3 upon increasing additions of PPA·2TBA in $CD_3OD$ c=concentration of free guest. The dashed lines represent 1 and 2 equivalents of added guest with respect to host. (B) Job's plot corresponding to the system in A.

A.

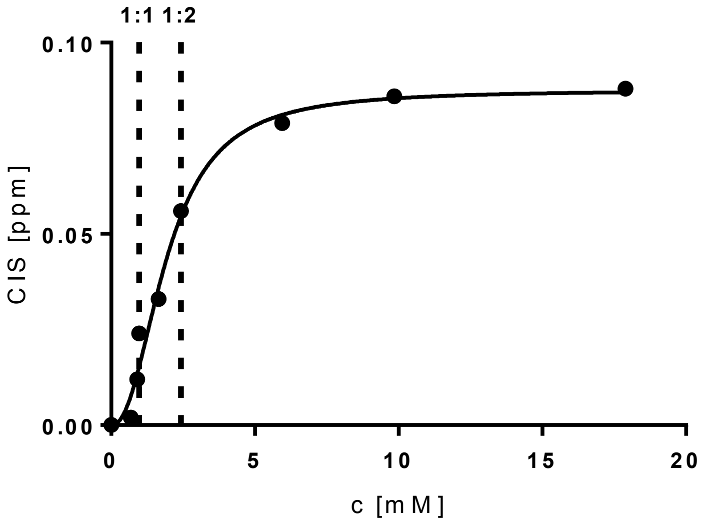


B.

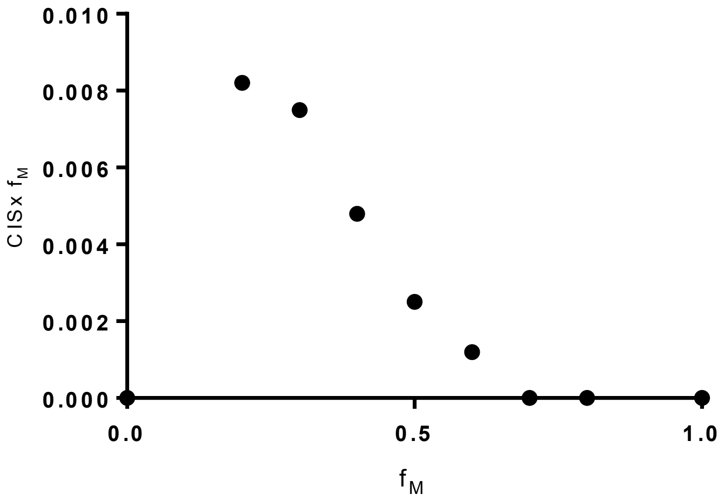


Figure S4. (A) $^1$H NMR titration curves showing the complexation induced shifts (CIS) of diagnostic protons of 4 upon increasing additions of PPA·2TBA in $CD_3OD$ c=concentration of free guest. The dashed lines represent 1 and 2 equivalents of added guest with respect to host. (B) Job's plot corresponding to the system in A.

A.

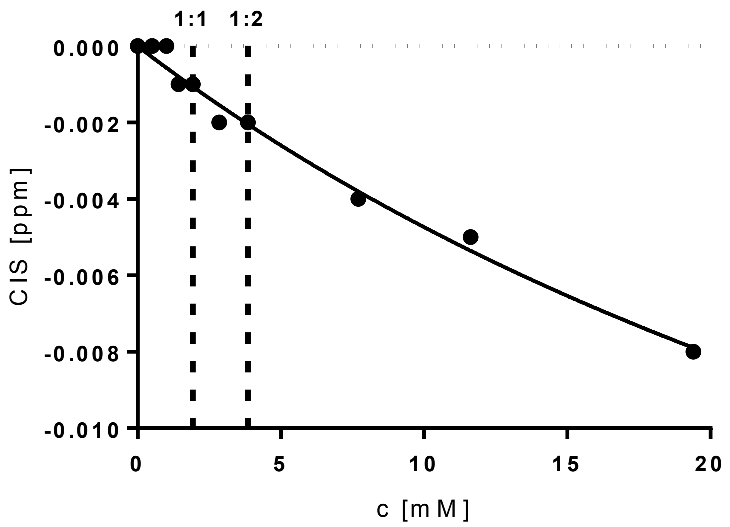


B.

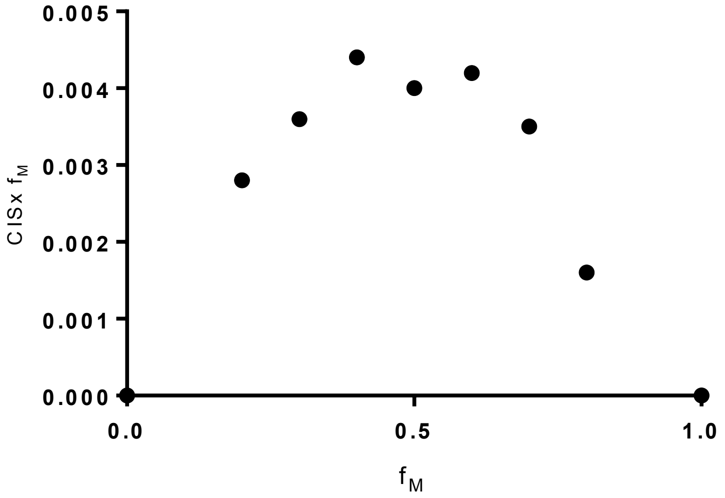


Figure S5. (A) $^1$H NMR titration curves showing the complexation induced shifts (CIS) of diagnostic protons of 5 upon increasing additions of PPA·Na in $CD_3OD$, c=concentration of free guest. The dashed lines represent 1 and 2 equivalents of added guest with respect to host. (B) Job's plot corresponding to the system in A.

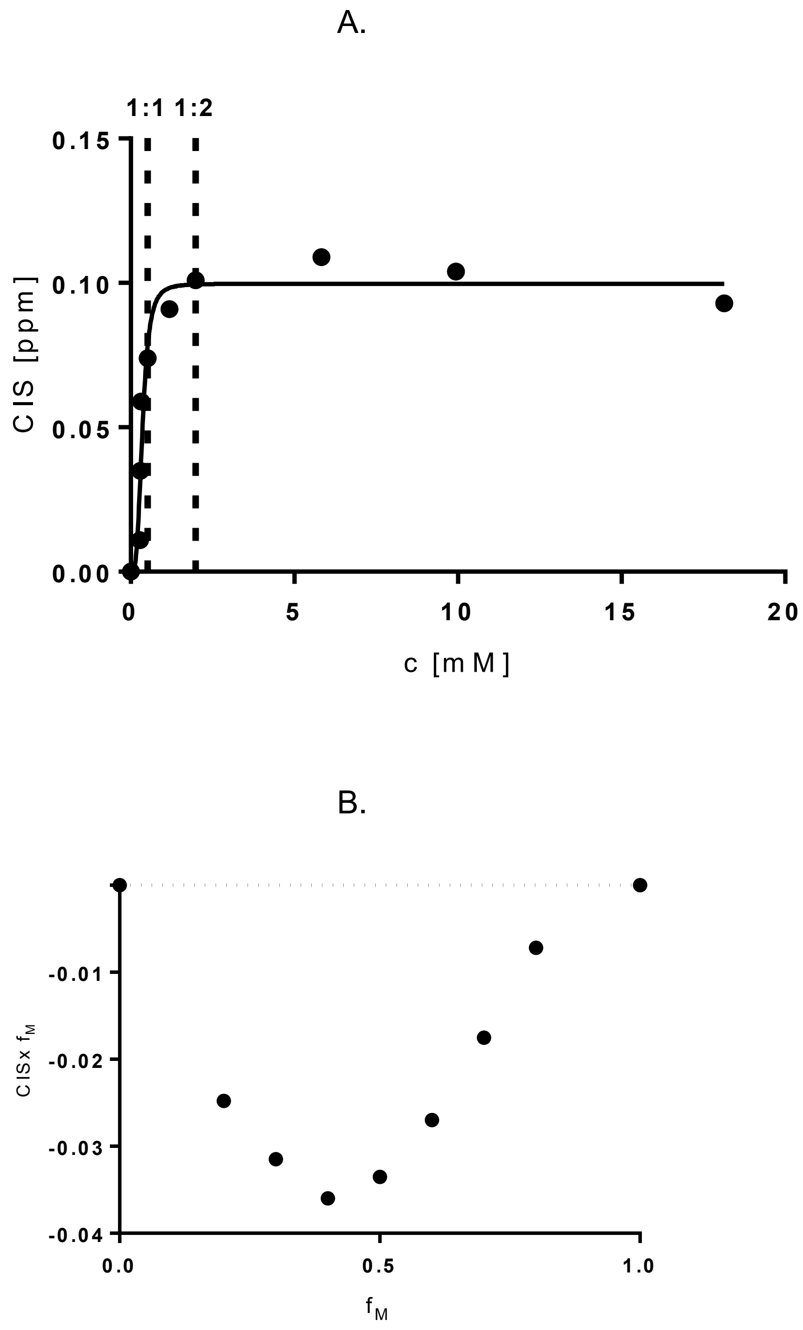


Figure S6. (A) $^{1}$H NMR titration curves showing the complexation induced shifts (CIS) of diagnostic protons of 5 upon increasing additions of PPA·2Na in $CD_3OD$ c=concentration of free guest. The dashed lines represent 1 and 2 equivalents of added guest with respect to host. (B) Job's plot corresponding to the system in A.

A.

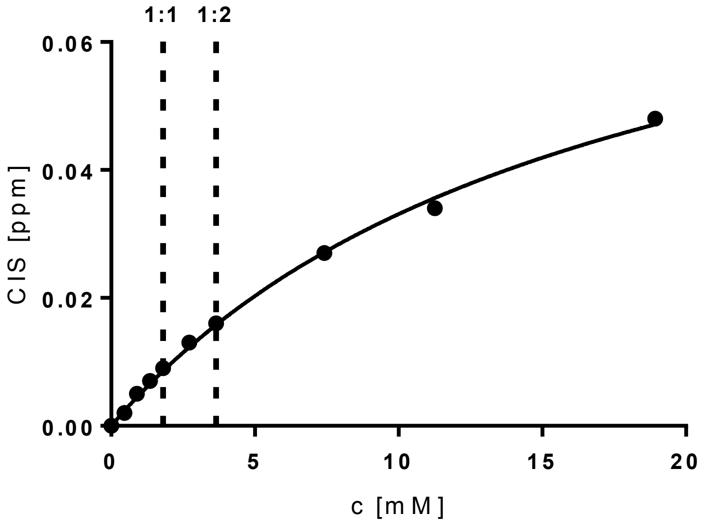


B.

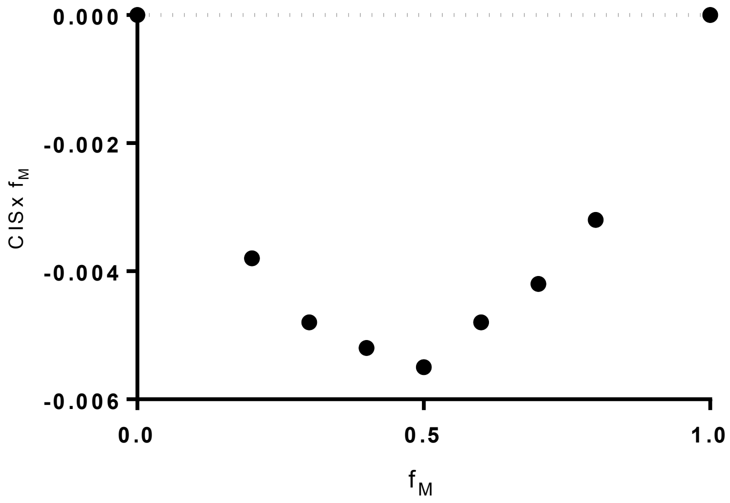


Figure S7. (A) $^{1}$H NMR titration curves showing the complexation induced shifts (CIS) of diagnostic protons of 5 upon increasing additions of PPA·2PMP in $CD_3OD$ c=concentration of free guest. The dashed lines represent 1 and 2 equivalents of added guest with respect to host. (B) Job's plot corresponding to the system in A.

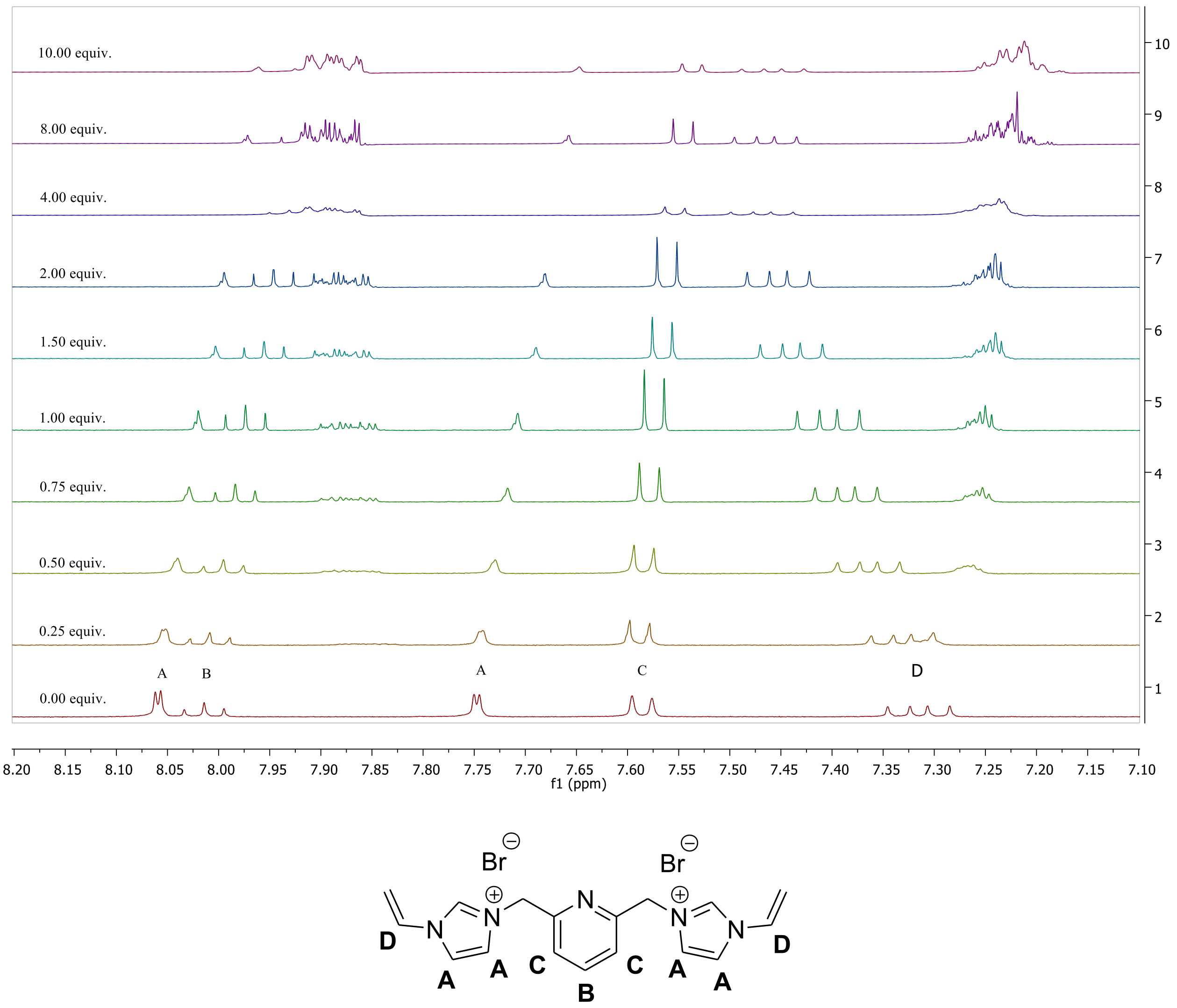


Figure S8. $^{1}$H NMR spectra of 5 upon increasing additions of up to 10 equivalents of PPA·2TBA in $CD_3OD$.

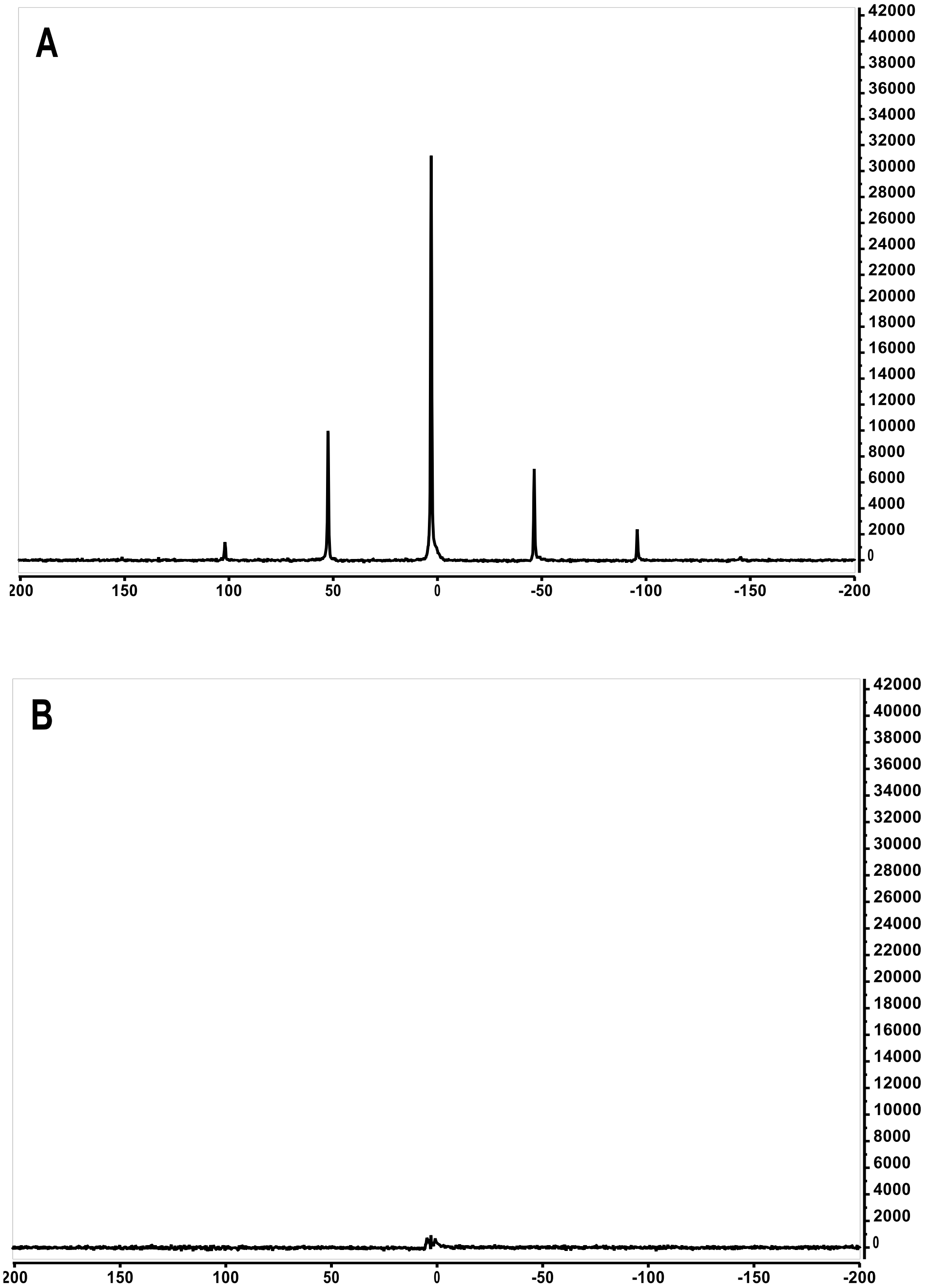


Figure S9. A) Solid state $^{31}$P NMR of a PA imprinted polymer prepared similarly to P8 before soxhlet extraction with methanol (extra side peaks are from $^{13}$C coupling) B) Solid state $^{31}$P NMR of the same polymer after soxhlet extraction with methanol (y-axis is the same scale, x-axis is δ in ppm)

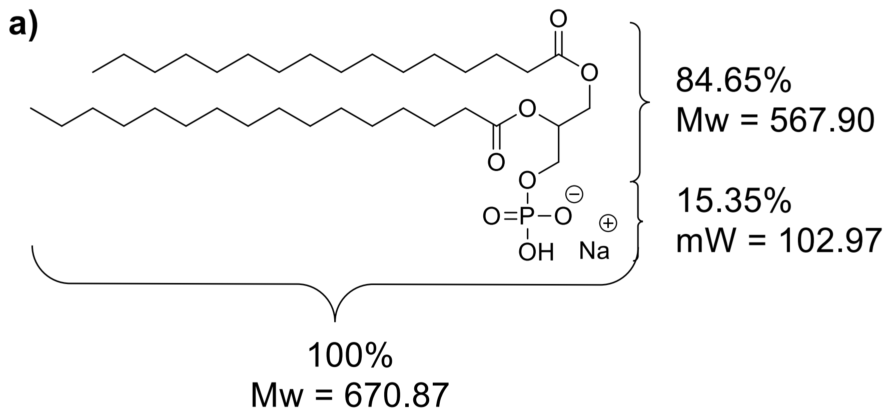


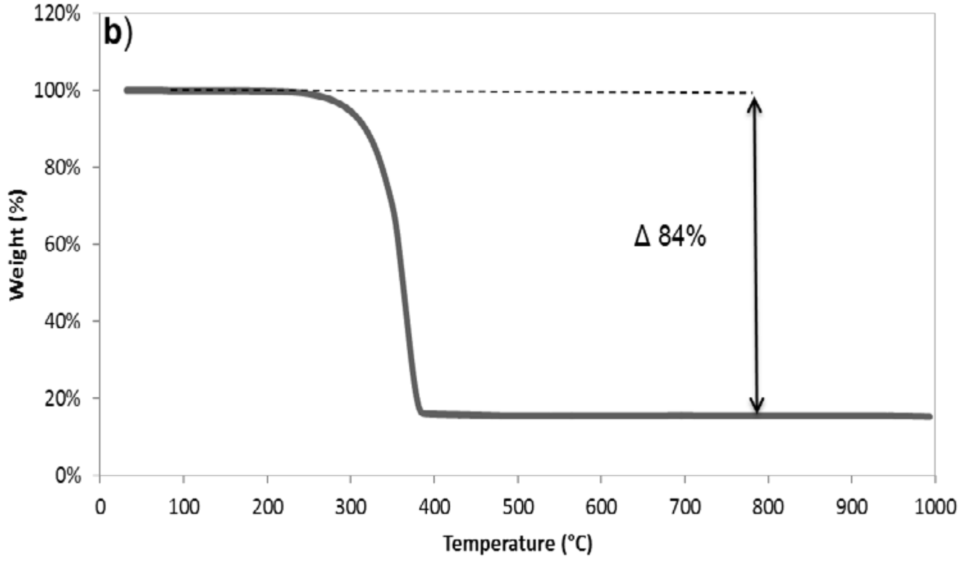


Figure S10. a) Molecular weights of fragments of PA. b) Thermal gravimetric analysis (TGA) of PA.

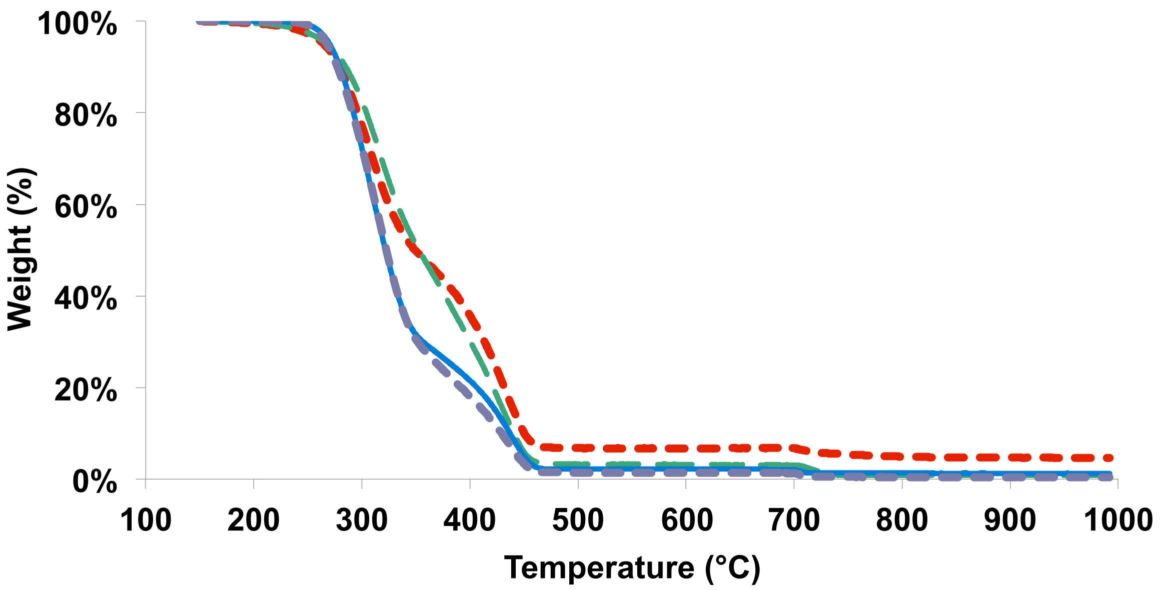


A.

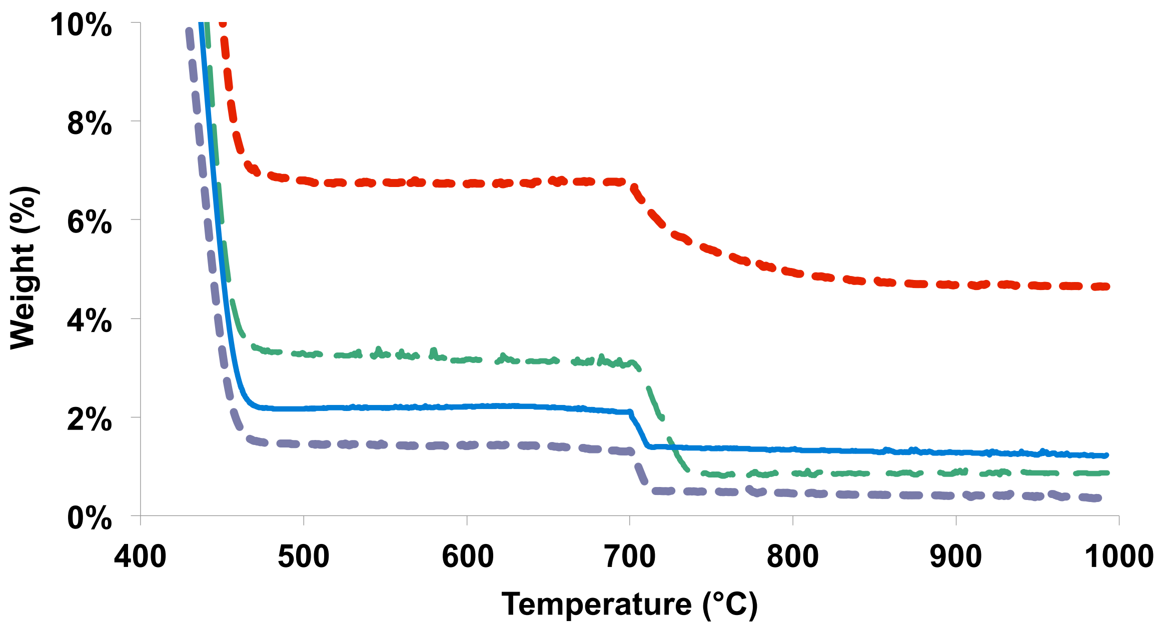


B.

Figure S11. TGA of a PA imprinted polymer prepared similarly to P8 before soxhlet extraction (big red dots) and after Soxhlet extraction (small blue dots) and a corresponding nonimprinted polymer before (green dashed lines) and after (purple dashed lines) soxhlet extraction.

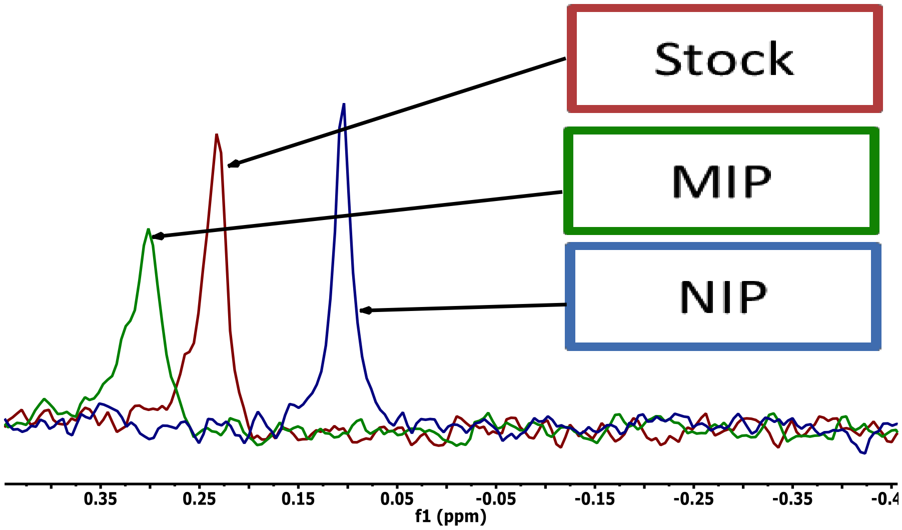


Figure S12. Comparison of $^{31}P$ NMR of a PA standard 3mg/mL and the supernatant isolated after incubation with a PA imprinted polymer (MIP) and nonimprinted polymer (NIP) in $CHCl_3$/methanol/$H_2O$ (30/10/5). All peaks were normalized to $Ph_4PCl$ used as internal standard.

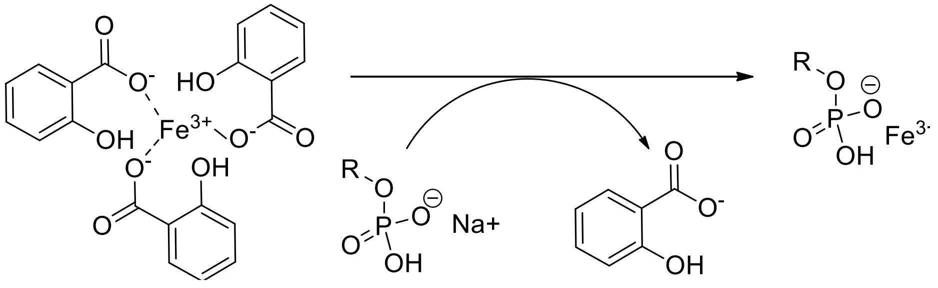


Figure S13. The Fe(III)$Sal_3$ colorimetric phosphate assay based on the discoloration of the Fe(III)$Sal_3$ complex by ligand displacement induced by phosphate. (J. Am. Oil. Chem. Soc. 2010, 87,1005)
